# Transcanal endoscopic horizontal semicircular canal occlusion for Meniere’s disease: A case series of 4 patients

**DOI:** 10.1097/MD.0000000000043296

**Published:** 2025-07-04

**Authors:** Mengwen Shi, Renhong Zhou, Yangming Leng, Enhao Wang, Yongqin Li, Jintao Yu, Le Xie, Qinming Cai, Yu Sun

**Affiliations:** a Department of Otorhinolaryngology, Union Hospital, Tongji Medical College, Huazhong University of Science and Technology, Wuhan, Hubei Province, China; b Institute of Otorhinolaryngology, Union Hospital, Tongji Medical College, Huazhong University of Science and Technology, Wuhan, Hubei Province, China; c Hubei Province Clinic Research Center for Deafness and Vertigo, Wuhan, Hubei Province, China.

**Keywords:** endoscopic ear surgery, refractory Meniere disease, semicircular canal occlusion, vertigo

## Abstract

**Rationale:**

Semicircular canal occlusion (SCO) is used to treat vestibular vertigo with demonstrated efficacy. This study introduces a novel transcanal endoscopic approach for horizontal SCO to manage vertigo in refractory Meniere’s disease (MD).

**Patient concerns:**

Four patients with refractory MD, previously managed conservatively for over 6 months, presented with severe-to-profound hearing loss. Their vertigo symptoms progressively worsened and severely compromised quality of life.

**Diagnoses:**

All 4 patients met diagnostic criteria for definite MD with severe-to-profound hearing loss.

**Interventions:**

All patients underwent transcanal endoscopic horizontal SCO with a 12-month postoperative follow-up to evaluate clinical outcomes and procedural safety.

**Outcomes:**

Postoperative vertigo control was effective, demonstrating significant reductions in both attack frequency and symptom intensity. Subjective tinnitus improved in 1 patient and remained stable in the remaining 3. Hearing thresholds were preserved in all cases without progression. Transient postoperative disequilibrium occurred in 1 patient, resolving spontaneously without complications.

**Lessons:**

Transcanal endoscopic horizontal SCO represents a safe, effective, and clinically viable alternative to pharmacologic conservative management for refractory MD with severe-to-profound hearing loss persisting beyond 6 months.

## 1. Introduction

**Figure 1.**
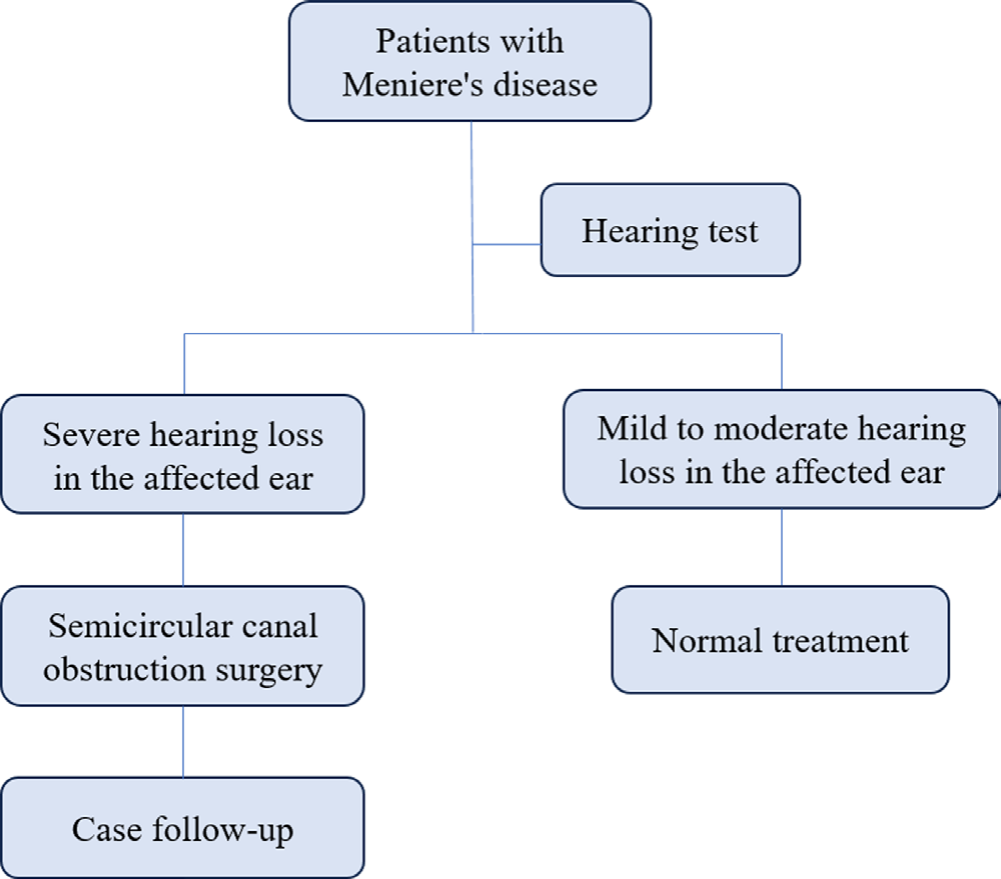
Flowchart of the study.

Meniere’s disease (MD) is distinguished by recurrent rotational vertigo, fluctuating sensorineural hearing loss, and tinnitus, occasionally accompanied by aural fullness.^[1]^ These symptoms significantly impair patients’ quality of life. The primary management goals are reducing attack frequency, alleviating symptoms (vertigo and tinnitus), and preserving residual hearing.^[2]^ When conservative therapies fail in persistent refractory MD, surgical intervention becomes viable. By converting dynamic bilateral vestibular excitation asymmetry into static asymmetry, surgery activates central compensatory mechanisms to alleviate vertigo.^[3]^ Semicircular canal occlusion (SCO) creates a bony window in the semicircular canal to obstruct endolymph flow and otolithic stimulation to the cupula utilizing bone wax, bone chips, peritoneum, biological glue, or laser technology.^[4]^ In 1990, Parnes et al^[5]^ first implemented transmastoid lateral SCO for refractory benign paroxysmal positional vertigo (BPPV), with subsequent studies validating its efficacy. Given that both MD and BPPV involve vertigo attacks mediated partially or entirely by endolymphatic fluid flow, SCO has seen a surge in clinical application for patients with refractory MD in recent years.^[6]^ Endoscopic techniques, being minimally invasive, have garnered widespread acceptance among patients by accessing middle/inner ear through the ear canal without invisible incision.^[7,8]^ This study (Fig. 1) achieves transcanal endoscopic horizontal SCO for the treatment of MD, expanding the application of endoscopic techniques in vertigo surgery.

## 2. Materials and methods

This study included 4 patients with refractory MD treated at our hospital from November 2019 to October 2023, including 1 male and 3 female patients, with an average age of (61.75 ± 10.01) years and a disease duration of 2 to 4 years.

Inclusion criteria are as follows:

1. Diagnosed as definite MD (the diagnostic criteria are as follows), accompanied by severe-to-profound hearing loss (hearing threshold in the affected ear >65 dB)^[9,10]^;
  - Two or more spontaneous episodes of vertigo, each lasting 20 min to 12 h.
  - Audiometrically documented low- to mid-frequency sensorineural hearing loss in 1 ear, defining the affected ear on at least 1 occasion before, during, or after 1 of the episodes of vertigo.
  - Fluctuating aural symptoms (hearing, tinnitus, or fullness) in the affected ear.
  - Not better accounted for by another vestibular diagnosis.
2. Standardized conservative treatment of MD for at least 6 months with frequent episodes of vertigo;
3. Patients have a strong desire for surgical treatment and fully understand and consent to treatment plans and possible risks.

Exclusion criteria are as follows:

1. Bilateral MD;
2. Central nervous system disorders or concurrent inner ear disorders;
3. Comorbidities requiring chronic diuretics/glucocorticoids therapy.

This study adheres to the principles of the Helsinki Declaration and has been approved by the Ethics Committee of Tongji Medical College, Huazhong University of Science and Technology (Ethics approval number: [2023]0602-01). All patients have provided written informed consent for participating in this study and publishing their case details.

### 2.1. Preoperative evaluation

All patients underwent comprehensive preoperative assessment comprising detailed medical history (Table 1), pure-tone threshold average, high-resolution temporal bone computed tomography, and endoscopic evaluation of the minimal external auditory canal diameter and tympanic membrane status. It is critical to confirm that patients meet the inclusion criteria with adequate anatomical dimensions for endoscopic otoscopy.^[11]^

**Table 1:** Information and symptoms of patients.

Patient	Age (years)	Gender	Duration (years)	Preoperation			Postoperative (follow-up more than 6 months)			
				Vertigo	Hearing (diseased ear)	Tinnitus and/or ear fullness	Vertigo	Hearing (diseased ear)	Tinnitus and/or ear fullness	Adverse reaction
Case 1	64	Male	4	Recurrent vertigo with no relief after 1 year of tympanic injection of dexamethasone.	70 dB HL	Persistent tinnitus with ear fullness	No attack	No progress	No paroxysmal exacerbation	No special discomfort
Case 2	63	Female	3	Frequent episodes, each lasting more than half an hour. The patient had been suffering from paroxysmal vertigo for more than 20 days before the surgery.	73 dB HL	Persistent tinnitus, patient denies ear fullness	No attack	No progress	No paroxysmal exacerbation	No special discomfort
Case 3	72	Female	2	Attacks occur 1–2 times a week, each lasting more than an hour.	deaf	Persistent, progressive tinnitus with ear fullness	No attack	No progress	Almost controlled, each tinnitus < 1 min	The patient has an unsteady gait and needs to walk with the help of a cane.
Case 4	48	Female	3	Recurrent paroxysmal vertigo, lasting more than half an hour each time, with no effect of medication.	75 dB HL	Fluctuating tinnitus, occasional ear fullness	No attack	No progress	No paroxysmal exacerbation	The patient is mildly dizzy, but does not affect life or work.

HL = hearing level.

### 2.2. Surgical procedures

**Figure 2.**
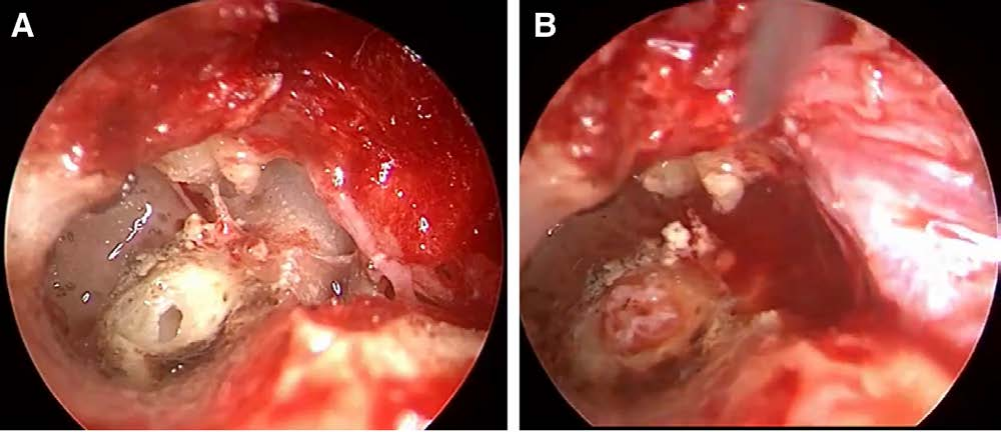
Fenestration of horizontal semicircular canal (A) and subcutaneous tissue tamponade of external auditory canal (B).

The preoperative preparation followed standard endoscopic ear surgery. The transcanal horizontal SCO surgery comprised these steps^[12,13]^:

1. Clean the external ear canal and inject freshly prepared lidocaine epinephrine solution subcutaneously to reduce bleeding during the preparation of the tympanic membrane flap;
2. Create a U-shaped tympanic membrane flap starting at the 3 o’clock position in the external auditory canal (>180° arc to prevent canal tissue tearing during elevation);
3. Enter the middle ear and dissect the malleus-incus joint. This step emphasizes the protection of the chorda tympani nerve and the stipe of the malleus;
4. Use a curette to scrape off a portion of the lateral wall of the epitympanum;
5. Enlarge the depression using a U-shaped bone to optimize middle ear exposure;
6. Expose and dissect the incudostapedial joint;
7. Remove the incus;
8. Continue to scrape off a portion of the inferior wall of the epitympanum to expose mesotympanum and hypotympanum;
9. Identify the horizontal semicircular canal and carefully create a bony window with a drill, taking care to avoid damaging the membranous labyrinth;
10. Use a hook needle to enlarge the bony window, exposing the membranous semicircular canal;
11. Compress the membranous semicircular canal against the contralateral bony wall using external auditory canal subcutaneous tissue, achieving complete endolymphatic flow obstruction (Fig. 2);
12. Achieve hemostasis with cotton pledgets. Seal the bony semicircular canal with bone wax, and cover it with absorbable hemostatic material if necessary;
13. Repair tympanic cavity defects with bone chips/powder;
14. Reposition the external auditory canal-tympanic membrane flap.

### 2.3. Postoperative follow-up

To assess postoperative ear wound healing at 2 weeks, membranous labyrinth MRI was performed. This evaluated horizontal SCO extent and surgical efficacy in the operated ear. At the 1-year follow-up, patients were interviewed regarding the frequency and severity of vertigo attacks, tinnitus, and ear fullness.

## 3. Results

**Figure 3.**
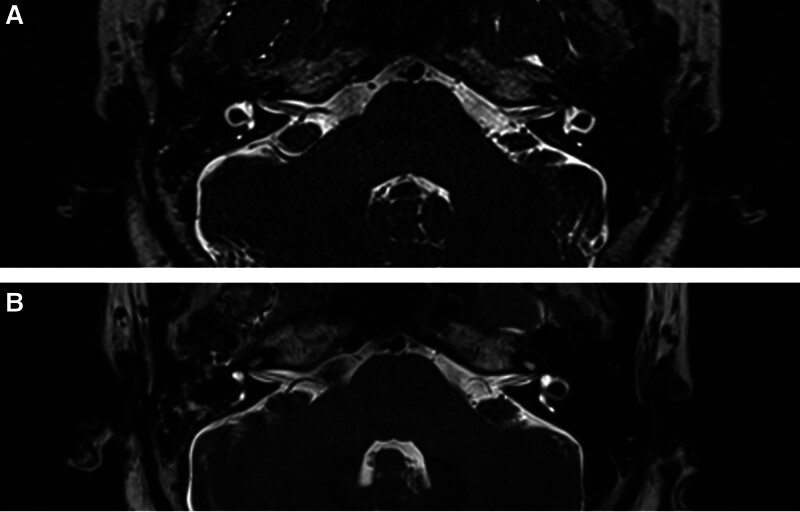
Magnetic resonance imaging (T2W_TSE) of the inner ear. (A) Preoperative. (B) Two weeks after surgery. T2W_TSE = T2-weighted turbo spin echo.

**Figure 4.**
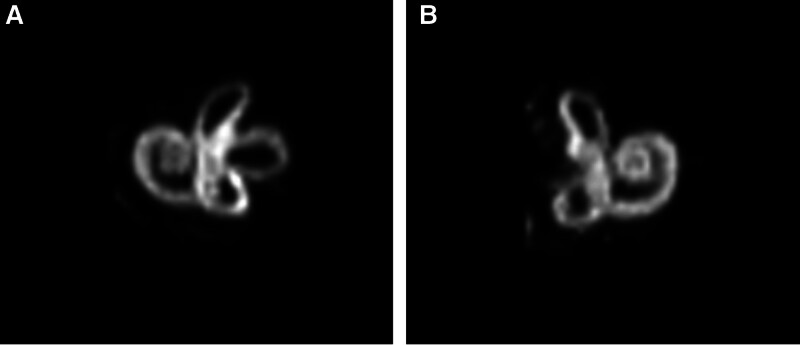
Three-dimensional magnetic resonance imaging (T2W_TSE) of the membranous labyrinth after horizontal semicircular canal occlusion. (A) Healthy ear. (B) Surgical ear. T2W_TSE = T2-weighted turbo spin echo.

As of November 2024, patient follow-up data are presented in Table 1. Postoperative imaging at two weeks confirmed 100% horizontal SCO (Figs. 3 and 4). No patients experienced short-term surgical complications such as surgical site infection, hemorrhage, severe pain, facial paralysis, or ageusia. At 1-year follow-up:

- All 4 patients remained vertigo-free;
- No bilateral hearing loss occurred;
- Tinnitus improved in one patient, while the others did not report worsening of tinnitus;
- One patient reported transient disequilibrium;
- No patients developed tympanic membrane perforation, otorrhea, or chronic pain.

## 4. Discussion

Our research has shown that occlusion of the horizontal semicircular canal via the external auditory canal effectively alleviated vertigo symptoms among patients suffering from MD, with no recurrence of vertigo or persistent long-term complications observed during a 1-year follow-up period.

The underlying pathology of MD involves endolymphatic effusion within the inner ear, with vertigo arising when potassium-rich endolymph escapes into the sodium-rich perilymph.^[14,15]^ By achieving permanent and complete occlusion of the semicircular canal, the flow of endolymph is effectively blocked. This, in turn, controls vertigo by eliminating vestibular stimuli and minimizing sensitivity to rotational acceleration of the head.^[16,17]^ SCO stands as an excellent surgical treatment for MD, with a remarkable success rate in controlling vertigo. The SCO procedure involves the occlusion of one to 3 semicircular canals. Among them, the triple semicircular canal occlusion (TSCO) is the most widely used and has the highest rate of vertigo control. A study followed 200 patients with MD who were treated with TSCO. The short-term vertigo control rate was 100%, and among the 49 patients followed up for more than 3 years, the vertigo control rate remained at 100%, with approximately 30% experiencing hearing loss.^[18,19]^ Compared with traditional MD surgical procedures (such as endolymphatic sac surgery, vestibular nerve section, and labyrinthectomy), TSCO has the following advantages: higher vertigo control rate and hearing preservation rate, better protection of otolith function, shorter postoperative imbalance time, and faster establishment of central compensation, thus it has been widely applied.^[20]^

However, it is noteworthy that some MD patients may experience compromised vestibular function and/or enduring hearing loss after SCO during long-term monitoring.^[21–24]^ A retrospective study reported that the incidence of permanent hearing loss was approximately 16.7%, and the incidence of vestibular function impairment was about 10%.^[25]^ The study revealed that following triple SCO procedures, the probability of additional hearing loss in the affected ear was approximately 30%. Comparatively, the probabilities were 23.8% and 20% after undergoing twice and single SCO procedures, respectively.^[4]^ Wang et al^[26]^ conducted occlusion of the posterior and horizontal semicircular canals in mice, and their findings revealed that auditory function, hair cell morphology, and endocochlear potential remained unaffected in both ears 4 weeks postsurgery. These results offer a compelling theoretical basis for applying this procedure to patients, even those with normal hearing. In the context of BPPV, posterior SCO is recognized as a safe and efficacious intervention, consistently achieving high levels of patient satisfaction.^[25,27]^ Compared to procedures involving multiple SCOs, the balance disorder response following horizontal SCO is generally less severe, and vestibular central compensation can be established more rapidly. SCO induces a sustained physical stimulus, resulting in a substantial increase in the concentrations of the excitatory neurotransmitter glutamate and the neuromodulator 5-hydroxytryptamine within the ipsilateral vestibular nucleus, which facilitates the process of vestibular compensation.^[28,29]^ Animal studies have further corroborated that unilateral horizontal SCO elevates 5-hydroxytryptamine levels within the central vestibular neural network, the duration of imbalance is shortened, and the time frame necessary for the establishment of vestibular center compensation is not only reduced but also more comprehensive.^[30]^ In cases of head-jolting nystagmus, Adolfo et al^[31]^ reported successful alleviation of vertigo through occlusion of the horizontal semicircular canal on the side of the fast phase. Subsequently, Welgampola et al^[32]^ further demonstrated that horizontal SCO could also relieve vertigo, albeit with the trade-off of hearing loss in the operated ear. The primary origin of eye movements lies in the horizontal semicircular canals.^[33]^ Building upon these studies, we implemented horizontal SCO specifically for patients with severe hearing loss.

For patients with refractory MD with severe hearing loss, SCO may be the preferred surgical option, when strict surgical indications are met. Clinically, corticosteroid injections into the middle ear constitute a secondary treatment alternative for MD, while tympanic injection of gentamicin serves as a bridge between secondary and tertiary surgical interventions.^[34,35]^ Surgery is considered for persistent vertigo, severely compromised quality of life, and medication-refractory cases. Surgical indications encompass stage IV unilateral MD or stage III with unsuccessful endolymphatic sac surgery, a speech recognition rate below 50%, and a patient’s strong inclination towards surgical intervention.^[36]^ The transmastoid SCO is efficient and safe but requires extensive mastoidectomy via postauricular incision, leading to increased postoperative symptoms such as pain and prolonged numbness.^[37–39]^ Endoscopic surgery obviates the need for postauricular incisions and extensive mastoid bone removal, improving patient satisfaction by avoiding head bandages, pain, and prolonged bed rest.^[12]^ Our transcanal SCO criteria align with traditional transmastoid indications. When there is no functional hearing in the affected ear, transcanal endoscopic surgery is more suitable. Therefore, if residual hearing remains in the affected ear, exercise extreme caution to protect the ossicular chain and other adjacent structures, and install artificial ossicles if necessary. Studies have demonstrated that the obstruction of either the superior or posterior semicircular canal alone can impact the entire vestibular system and hearing. Notably, the duration and extent of recovery vary among patients, with the precise underlying cause remaining unknown.^[23]^ Global researchers have proposed the creation of disease progression prediction models leveraging imaging techniques. Bilateral progression risk generally contraindicates surgery.^[40,41]^ Consequently, before contemplating surgical intervention, it is imperative to (1) confirm the diagnosis and the affected side, (2) evaluate the contralateral vestibular function, (3) exhaust all available conservative treatment options, and (4) conduct personalized risk-benefit counseling.

In our study, the transcanal approach, although mini-invasive, necessitates the removal of the incus, creating conductive hearing loss. However, given the hearing status clearly outlined in the paper, with patients having hearing thresholds over 70 dB, the extent of this comorbidity remains acceptable to the patients. Postoperative transient imbalance, nausea, and vomiting are common symptoms after SCO surgery, and usually recover within two weeks. During follow-up, 1 patient was found to have significantly improved symptoms of vertigo, and the tinnitus disappeared, but she had residual symptoms of unsteady gait. We found that she had not undergone vestibular rehabilitation as directed postoperatively, which, in addition to the surgical factors, may have been the main cause of the patient’s poor vestibular function.^[42,43]^ Furthermore, the patients were older and exhibited more severe symptoms before surgery, which could potentially have influenced their vestibular function recovery postoperatively.^[44,45]^ Consequently, we hypothesize that a combination of precise surgical intervention and early initiation of vestibular rehabilitation may be the optimal approach to ensure treatment effectiveness.

## 5. Conclusion

In summary, the transcanal endoscopic approach for horizontal SCO is a safe, effective, and well-tolerated treatment option. It serves as a viable alternative for patients with MD who have undergone conservative treatment for over 6 months without significant improvement and have severe-to-profound hearing loss.

## Acknowledgments

The authors thank the patients for their participation in this study.

## Author contributions

**Investigation:** Mengwen Shi, Yangming Leng, Jintao Yu.

**Writing – original draft:** Mengwen Shi, Qinming Cai.

**Writing – review & editing:** Mengwen Shi, Yu Sun.

**Validation:** Renhong Zhou, Yangming Leng, Yongqin Li.

**Methodology:** Yangming Leng, Enhao Wang, Yu Sun.

**Conceptualization:** Jintao Yu, Yu Sun.

**Data curation:** Le Xie.

**Funding acquisition:** Yu Sun.

**Project administration:** Yu Sun.

**Supervision:** Yu Sun.
